# Effects of Low Energy Availability on Reproductive Functions and Their Underlying Neuroendocrine Mechanisms

**DOI:** 10.3390/jcm7070166

**Published:** 2018-07-05

**Authors:** Takeshi Iwasa, Toshiya Matsuzaki, Kiyohito Yano, Yiliyasi Mayila, Rie Yanagihara, Yuri Yamamoto, Akira Kuwahara, Minoru Irahara

**Affiliations:** Department of Obstetrics and Gynecology, Institute of Biomedical Sciences, Tokushima University Graduate School, 3-18-15 Kuramoto-Cho, Tokushima 770-8503, Japan; iwasa1976@gmail.com (T.M.); yano.kiyohito@tokushima-u.ac.jp (K.Y.); maira2309@yahoo.co.jp (Y.M.); yanagihara.rie@tokushima-u.ac.jp (R.Y.); yamamoto.yuri@tokushima-u.ac.jp (Y.Y.); ch.kurahara@tokushima-u.ac.jp (A.K.); irahara.minoru@tokushima-u.ac.jp (M.I.)

**Keywords:** undernutrition, GnRH, leptin, NPY, AgRP, kisspeptin

## Abstract

It is known that metabolic disturbances suppress reproductive functions in females. The mechanisms underlying metabolic and nutritional effects on reproductive functions have been established based on a large body of clinical and experimental data. From the 1980s to 1990s, it was revealed that disrupted gonadotropin-releasing hormone (GnRH) secretion is the main cause of reproductive impairments in metabolic and nutritional disorders. From the late 1990s to early 2000s, it was demonstrated that, in addition to their primary functions, some appetite- or metabolism-regulating factors affect GnRH secretion. Furthermore, in the early 2000s, kisspeptin, which is a potent positive regulator of GnRH secretion, was newly discovered, and it has been revealed that kisspeptin integrates the effects of metabolic status on GnRH neurons. Recent studies have shown that kisspeptin mediates at least some of the effects of appetite- and metabolism-regulating factors on GnRH neurons. Thus, kisspeptin might be a useful clinical target for treatments aimed at restoring reproductive functions in individuals with metabolic or nutritional disturbances, such as those who exercise excessively, experience marked weight loss, or suffer from eating disorders. This paper presents a review of what is currently known about the effects of metabolic status on reproductive functions and their underlying mechanisms by summarizing the available evidence.

## 1. Introduction

It is known that a lack of metabolic fuel suppresses reproductive functions in females, e.g., it results in puberty being delayed, chronic anovulation, and/or menstrual disorders [1,2,3,4]. Eating disorders, excessive exercise, and weight loss due to calorie restriction frequently induce amenorrhea or irregular menses [1,2,3,4], and they also cause reductions in bone mineral density and increase the risk of osteoporosis [5,6,7]. Recently, cardiovascular complications have been considered the causal substrate for the poor prognosis of anorexia nervosa [8]. In addition, some mental health problems, such as social anxiety disorder and attention deficit/hyperactivity disorder, are commonly comorbid in eating disorders. These data indicate that undernutrition induces several negative health consequences including reproductive dysfunctions [9,10]. Interestingly, reproductive functions are also suppressed when energy stores cannot be properly utilized due to metabolic disturbances, such as diabetes or obesity, even when enough energy is being stored [11,12,13]. The mechanisms underlying metabolic and nutritional effects on reproductive functions have been well established based on a large body of clinical and experimental data. Because secretion of GnRH from hypothalamic neurons is difficult to measure, especially in human, most studies measured serum luteinizing hormone (LH) levels as an index for GnRH secretion. Thus, pulsatile secretion of LH reflects the pulsatile secretion of GnRH, whereas surge secretion of LH reflects GnRH surge. From the 1980s to 1990s, it was revealed that disruption of pulsatile gonadotropin-releasing hormone (GnRH) secretion from the hypothalamus is the main cause of the reproductive impairments induced by metabolic and nutritional disorders [14,15,16,17]. From the late 1990s to early 2000s, it was demonstrated that, in addition to their primary functions, some appetite- and metabolism-regulating factors affect GnRH secretion [18]. Furthermore, in the early 2000s, kisspeptin, which is a potent positive regulator of GnRH secretion, was newly discovered, and subsequent studies have shown that kisspeptin integrates the effects of metabolic status on GnRH neurons [19,20]. This paper presents a review of what is currently known about the impact of negative energy balance on reproductive functions and the mechanisms underlying these effects by summarizing the available evidence.

## 2. Effects of Nutrition on Reproductive Functions

In 1980, it was reported that the reproductive function of young female dancers was disrupted by a lack of energy [21]. Menarche was markedly delayed in the dancers, who maintained a high level of physical training from early adolescence onwards. Although the dancers’ sexual development progressed, and menarche occurred, after their exercise schedule was reduced or in periods of forced rest caused by injuries, amenorrhea recurred after heavy exercise was restarted. In 1984, the concept of brain energy availability was proposed [22]. Namely, it was suggested that the brain monitors the balance between the availability and utilization of calories, and brain functions are altered when there is insufficient metabolic fuel available to meet the energy requirements of the brain. Such alterations in brain functions might cause the delayed onset of menarche and the reversible cessation of menses.

## 3. The Effects of a Negative Energy Balance on GnRH/LH Secretion

**Figure 1:**
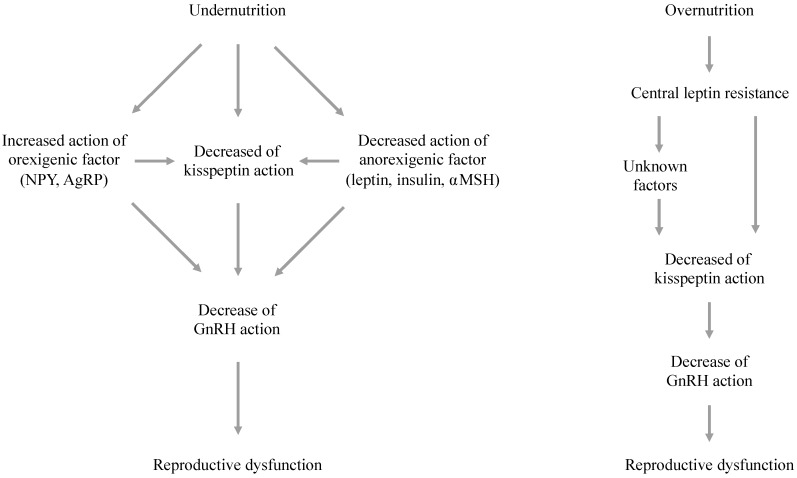
The roles of central and peripheral factors in reproductive dysfunction under under-nourished and over-nourished conditions. In addition to their primary functions, appetite- and metabolism-regulating factors; i.e., orexigenic and anorexigenic factors, suppress or activate GnRH secretion. Changes in the levels of these factors promote feeding behavior, whereas they coordinately suppress GnRH secretion and induce reproductive dysfunction in the presence of a negative energy balance. In addition, kisspeptin neurons are sensitive to metabolic status, and their activities are suppressed in the presence of a negative energy balance. Such changes also adversely affect GnRH secretion. It has been revealed that the effects of appetite- and metabolism-regulating factors on GnRH are partially mediated by kisspeptin neurons. In addition, overnutrition induces central leptin resistance and this alteration directly and indirectly decrease kisspeptin action on GnRH, and consequently induce reproductive dysfunction.

Reproductive functions are mainly regulated by the hypothalamic-pituitary-gonadal (HPG) axis; i.e., GnRH, gonadotropins, and gonadal steroids, in humans and animals. Among these factors, hypothalamic GnRH acts as a central regulator of the HPG axis (Figure 1). Previous studies have revealed that reductions in energy availability suppress HPG activity by inhibiting GnRH, thereby decreasing LH secretion from the pituitary. For example, the mean plasma LH levels of females with hypothalamic amenorrhea, whose symptoms were mainly caused by weight loss, were lower than those of normal females [14]. In addition, the LH pulse frequency was lower in females with hypothalamic amenorrhea than in normal females during the early follicular phase [14], indicating that persistent anovulation followed by menstrual abnormalities might be induced by reductions in the pulsatile secretion of GnRH. Interestingly, it was demonstrated that the pulsatile secretion of LH is abruptly disrupted when energy availability falls below a certain threshold, instead of decreasing linearly along with energy status [17]. I’anson et al. have simultaneously evaluated the GnRH and LH secretion by blood sampling from hypophyseal portal blood and jugular blood in female sheep and revealed that both frequency and amplitude of GnRH pulses are decreased in growth-restricted hypogonadotropic individuals [23]. Based on these results, pulsatile GnRH treatment has been used to induce ovulation in patients with hypothalamic amenorrhea, and favorable outcomes have been obtained with this method [24,25]. In other words, these clinical findings support the hypothesis that reduced pulsatile secretion of GnRH is the main cause of the reproductive dysfunctions induced by a negative energy balance.

## 4. Hormonal and Neuropeptide Pathways That Connect Metabolic Status and GnRH Neurons

Energy restriction caused decreased GnRH/LH pulsatility in some experimental animals, and the hormonal and neuroendocrine mechanisms underlying these alterations have been evaluated using animal models, as well as in humans. In these experiments, it was demonstrated that, in addition to their primary functions, some appetite- and metabolism-regulating factors affect GnRH/LH secretion (Figure 1). In general, satiety-related factors, e.g., leptin, insulin, pro-opiomelanocortin (POMC), and alpha-melanocyte-stimulating hormone (αMSH), directly or indirectly stimulate LH secretion, whereas orexigenic factors, e.g., neuropeptide Y (NPY), orexin, and ghrelin, suppress LH secretion [18]. Thus, reductions in the activity of these satiety-related factors and increases in the activity of orexigenic factors might suppress GnRH secretion and consequently cause reproductive dysfunction in the presence of a negative energy balance.

Leptin, which is an adipocyte-derived hormone, is involved in the regulation of appetite and reproductive functions. Leptin suppresses appetite and increases the metabolic rate, mainly through hypothalamic orexigenic and anorexigenic factors, and it also prevents excessive weight gain and the accumulation of fat [26,27,28]. In addition to these effects, leptin plays important roles in sexual maturation and fertility. Leptin-deficient *ob*/*ob* mice exhibited disturbances of puberty and infertility, and chronic leptin treatment increased the serum gonadotropin level and restored puberty and fertility in mice [29,30]. Similarly, chronic leptin administration accelerated the onset of puberty in normal female mice [31]. Furthermore, chronic leptin treatment normalized the serum gonadotropin level and restored estrous cyclicity in food-deprived female mice [32]. Although the effects of leptin on the HPG axis are primarily mediated through the stimulation of GnRH and gonadotropin secretion, GnRH neurons themselves do not have leptin receptors [33]. In addition, the ablation of the leptin receptor from all forebrain neurons prevented the onset of puberty and induced infertility in male and female mice, whereas selective deletion of the leptin receptor from GnRH neurons resulted in normal fertility [34]. These findings indicate that leptin does not act directly on GnRH neurons and that other forebrain neurons might be responsible for mediating the effects of leptin on GnRH. Insulin is involved in the regulation of GnRH secretion. It has been shown that neuron-specific disruption of the insulin receptor (IR) gene induces the reduction of serum LH levels and subsequent hypogonadism in female mice [35]. On the contrary, it has been reported that mice with selective ablation of IR on GnRH neurons display normal puberty and fertility [36]. These data indicate that insulin does not directly influence on GnRH neurons. Interestingly, mice with deletion of insulin like growth factor 1 (IGF1) showed low LH level and delayed pubertal development, indicating that IGF1 may directly affect the GnRH neurons [36].

POMC is a precursor protein which produces many biologically active peptides. In addition, POMC neurons within the hypothalamic ARC act as a vital anorexigenic factor, and both insulin and leptin excite their activities [37]. One brain region that POMC neurons project is the medial preoptic area (POA), where GnRH neurons are concentrated, and some of these neurons make synaptic contacts with GnRH neurons [38], indicating that POMC-derived peptides directly act on GnRH secretion. αMSH, which is a cleavage product of POMC gene, is an anorexigenic neuropeptide and it exerts its effect by binding with the melanocortin 4 receptor (MC4R) [39]. It has been shown that αMSH stimulates the GnRH system by acting on MC4R. GnRH neurons have MC4R [40] and central administration of αMSH increases the serum LH level [41]. In addition, MC4R deficient mice exhibit reduced ovulation rates and fertility [42]. Furthermore, normalization of melanocortin signaling improves the fertility in leptin receptor knock-out *db*/*db* mice [41], suggesting that melanocortin signaling mediates the leptin actions on GnRH neurons. 

NPY is a potent hypothalamic orexigenic factor [43]. Previous studies have shown that there is a pivotal link between NPY and GnRH neurons. NPY neurons are found in close contact with GnRH neurons and might directly input signals into GnRH cell bodies and nerve terminals through the NPY Y1 receptor [44]. The effects of NPY on LH secretion are affected by the gonadal steroidal environment. NPY inhibited LH secretion and reduced gonadotropin levels in ovariectomized female rats [45,46], whereas it stimulated GnRH and increased gonadotropin levels in intact rabbits and estradiol-primed ovariectomized rats under in vivo and in vitro conditions [47,48]. It has been reported that NPY is involved in the negative energy balance-induced suppression of GnRH/gonadotropin secretion and that some effects of leptin on GnRH/gonadotropin or appetite might be mediated by NPY neurons [49,50,51]. Food deprivation was found to increase hypothalamic NPY activity and gene expression while concomitantly reducing LH secretion [52]. The gonadotropin levels of NPY-deficient mice were not decreased by fasting [53]. In addition, in the absence of NPY, the obesity and sterility normally exhibited by leptin-deficient *ob*/*ob* mice were attenuated, indicating that NPY functions as a central effector of leptin deficiency [49]. Agouti-related peptide (AgRP), which is hypothalamic orexigenic factor, is co-expressed with NPY in the neuronal population found in the ARC. It has been shown that AgRP has an inhibitory effect on LH secretion in monkey and ablation of AgRP expressing neurons in *ob*/*ob* mice recovery fertility [54,55]. These data indicate that AgRP is also involved in the central effects of leptin deficiency.

## 5. The Effects of Energy Availability on Hypothalamic Kisspeptin Signaling

Kisspeptin, which is encoded by the *Kiss1* gene, is a hypothalamic peptide, which directly stimulates GnRH synthesis and release via its receptor, Kiss1r [56,57,58,59,60,61]. The population of kisspeptin neurons located in the arcuate nucleus (ARC) is considered to mediate the effects of negative feedback signaling by estrogen and to be implicated in the GnRH/LH pulse generator in several species [62,63,64,65]. In rodents, the population of kisspeptin neurons located in the anteroventricular periventricular nucleus (AVPV) is considered to mediate the effects of positive feedback signaling by estrogen on GnRH/LH release [62,63,66,67]. These findings indicate that kisspeptin might integrate the effects of estrogen on GnRH neurons. In non-rodent species, a rostral population of kisspeptin neurons reside in the POA and together with the ARC kisspeptin neurons mediate sex-steroid positive feedback [65].

It has been revealed that the kisspeptin-Kiss1r system is highly sensitive to metabolic and nutritional conditions (Figure 1). A negative energy balance was shown to have a negative impact on hypothalamic kisspeptin neurons in both adult and prepubertal rodents [20,68,69,70,71]. Fasting reduced hypothalamic *Kiss1* gene expression and delayed the onset of puberty in prepubertal female rats, whereas the administration of exogenous kisspeptin restored gonadotropin secretion and normalized the onset of puberty [72]. In addition, food deprivation prolonged the estrous cycle by reducing *Kiss1* gene expression, and downregulated gonadotropin levels in adult female rats [70,73]. Some studies have examined the effects of fasting on the distinct kisspeptin neuronal subpopulations in the AVPV and ARC. *Kiss1* gene expression in the ARC was reduced by fasting in intact and prepubertal female rats [70,74], whereas its expression in the AVPV was reduced by food restriction in ovariectomized female rats [73]. Similarly, Kiss1 mRNA expressions in ARC and POA in lean ovariectomized ewe were lower than those in normal-weight ewe [75], and the number of kisspeptin immunoreactive neurons in ARC in fasted lambs were significantly lower than those in fed lambs [76]. It has been reported that both a negative energy balance and overnutrition affect the hypothalamic kisspeptin-Kiss1r system. For example, hypothalamic *Kiss1* gene expression and gonadotropin levels were reduced in streptozotocin-induced diabetic male rats, and normal serum gonadotropin levels were restored by the administration of kisspeptin [77]. Similarly, hypothalamic *Kiss1* gene expression was reduced in female mice in which infertility had been induced via a high fat diet [78]. These findings indicate that the kisspeptin expressed on GnRH neurons integrates a range of metabolic inputs.

## 6. Mechanisms Responsible for Metabolic Effects on the Kisspeptin System

Although the exact mechanisms by which metabolic factors alter hypothalamic kisspeptin neurons are unknown, some peripheral and central factors might affect their activity (Figure 1). Among these factors, the actions of leptin on kisspeptin neurons and their underlying mechanisms of action have been established in detail. In addition, there is growing evidence that some hypothalamic appetite-regulating factors, such as AgRP/NPY, also affect the activity of kisspeptin neurons.

As noted above, although the positive effects of leptin on the HPG axis are mediated through the stimulation of GnRH/LH secretion, GnRH neurons themselves do not have leptin receptors [33]. The available data suggest that hypothalamic kisspeptin mediates the effects of leptin on GnRH neurons. It was demonstrated that the leptin receptor is expressed on kisspeptin neurons in ARC [79,80], and hypothalamic *Kiss1* mRNA expression was reduced by the downregulation of leptin activity [77,78,81,82]. In addition, the administration of leptin restored *Kiss1* mRNA expression in leptin-deficient *ob*/*ob* mice and diabetic rats [77,79,82]. These data suggest that decrease of leptin secretion from adipocyte may reduce hypothalamic *Kiss1* gene expression in undernourished condition and that these alterations may induce the decreased action of kisspeptin and subsequent reproductive dysfunctions. In contrast, Donato et al. have shown that selective genetic deletion of leptin receptor from hypothalamic Kiss1 neurons does not induce any effects on puberty and fertility in mice [83]. This data indicates that leptin’s action of reproductive function might not be mediated by kisspeptin neurons. Therefore, further examinations would be needed to clarify the relationship between leptin and kisspeptin in the regulation of reproductive functions. We should be cautious when interpreting congenital ablation studies, as cited above, as pompensation is always a possibility. For example, it has been shown that ablation of AgRP or NPY prior to birth induces little effect on body weight [49,84], while ablation of these factors after birth induces starve within days [85,86]. Thus, it is possible that such discrepancy would be occurred even in reproductive phenotypes. Interestingly, reduction of hypothalamic *Kiss1* gene expression is also observed in over-nourished obese individuals. Quennell and colleague have shown that hypothalamic *Kiss1* mRNA expressions in high-fat-diet induced obese female mice, which are prone to infertility, are significantly lower than those fed with standard diet [78]. On the contrary, serum leptin level in diet-induced obese mice is significantly higher than that in those fed with standard diet, indicating that diet-induced obese mice exhibit central resistance to leptin signaling of reproductive functions, as well as of metabolic functions. They also evaluated the molecular mechanism by which leptin act on the kisspeptin neurons, and showed that activation of leptin-signaling molecules, i.e., pSTAT3, pSTAT5, and pS6, after leptin injection did not occurred in kisspeptin-expressing cell in anterior part of hypothalamus [78]. This result indicates that the effects of leptin on the AVPV might be indirectly mediated by unknown upstream neurons, whereas its effects on the ARC may be direct. As noted above, NPY and AgRP are hypothalamic orexigenic factors. They are co-expressed in the neuronal population found in the ARC, and their expression is upregulated under negative energy balance conditions in order to stimulate feeding [87,88]. AgRP/NPY neurons are a direct target of leptin, and they play roles in leptin-associated infertility under negative energy balance conditions [69,70]. It has been reported that inhibitory synaptic connections exist between AgRP neurons and kisspeptin neurons and that kisspeptin neurons were subjected to less marked presynaptic suppression when AgRP neurons were ablated [89,90]. In addition, the activation of AgRP neurons was reported to prolong the estrous cycle and reduce fertility [69,70]. Furthermore, kisspeptin neurons express NPY receptors [89]. These findings indicate that AgRP/NPY neurons suppress GnRH secretion and subsequently reduce fertility, at least in part through the inhibition of kisspeptin activity.

## 7. Conclusions

GnRH secretion from the hypothalamus is decreased by metabolic and nutritional disorders, which results in reproductive impairments. It has been shown that some appetite- and metabolism-regulating factors directly or indirectly affect GnRH neurons, and that alterations in the levels of these factors suppress GnRH and gonadotropin secretion, as well as increase appetite and feeding behavior, in the presence of a negative energy balance. In addition, it has been clarified that kisspeptin integrates the effects of metabolic status on GnRH neurons and that kisspeptin mediates at least some of the effects of appetite- and metabolism-regulating factors on GnRH neurons. Therefore, kisspeptin might be a useful clinical target for treatments aimed at restoring reproductive functions in individuals who exercise excessively; have experienced marked weight loss; or have metabolic or nutritional disorders, such as eating disorders.
